# Cardiac sodium-glucose co-transporter 1 (SGLT1) contributes to heart failure in a mouse model of diabetic cardiomyopathy

**DOI:** 10.1007/s00395-025-01136-7

**Published:** 2025-09-11

**Authors:** Zhao Li, Sydney Freiberg, Meredith L. Music, Lina Gu, Sarah Nacos, Joseph P. Phillips, Adil Hassan, Kamel Shibbani, Sanah S. Munir, Vooha K. Kumar, Luke Halligan, Mia E. Michel, Benjamin F. London, Ngan Bui, Michael Cicha, Valerie Buffard, E. Dale Abel, Ferhaan Ahmad

**Affiliations:** 1 Division of Cardiovascular Medicine, Department of Internal Medicine, Carver College of Medicine and Abboud Cardiovascular Research Center, University of Iowa, 25 South Grand Avenue, 1191D ML, Iowa City, IA 52242 USA; 2 Department of Pharmaceutical Sciences, University of St. Joseph, West Hartford, CT USA; 3 Division of Endocrinology and Metabolism, Department of Internal Medicine, Carver College of Medicine and Abboud Cardiovascular Research Center, University of Iowa, Iowa City, IA USA; 4 Department of Medicine, David Geffen School of Medicine, University of California Los Angeles, Los Angeles, CA USA; 5 Department of Molecular Physiology and Biophysics, Carver College of Medicine, University of Iowa, Iowa City, IA USA; 6 Department of Radiology, Carver College of Medicine, University of Iowa, Iowa City, IA USA

**Keywords:** Cardiomyopathy, Diabetes, Fibrosis, Glucose, Insulin, Oxidative stress

## Abstract

Diabetes mellitus can lead to a cardiomyopathy independent of other risk factors such as coronary artery disease and hypertension, in up to 75% of patients. The prevalence of diabetic cardiomyopathy in the population is 1.1%. We previously showed that SGLT1 is expressed in cardiomyocytes and is further upregulated in diabetic cardiomyopathy and other forms of heart failure. In this study, we sought to determine the mechanisms by which cardiac SGLT1 contributes to the pathophysiology of heart failure in diabetes, obesity, and insulin resistance. We determined whether transgenic mice with cardiomyocyte-specific knockdown of SGLT1 (TG^SGLT1-DOWN^) had attenuation of cardiomyopathy after induction of obesity and insulin resistance by exposure to a high fat diet (HFD) from ages 8–28 weeks. TG^SGLT1-DOWN^ mice and wildtype (WT) littermates exhibited similar increases in body weight and blood glucose after exposure to HFD. Nevertheless, TG^SGLT1-DOWN^ mice exhibited attenuation of cardiomyopathy, manifested by less hypertrophy, systolic and diastolic dysfunction, fibrosis, nicotinamide adenine dinucleotide phosphate oxidase 2 (Nox2) activation, and reactive oxygen species (ROS) production. In vivo hyperinsulinemia and in vitro exposure of cardiomyocytes to high glucose or insulin led to an increase in SGLT1 expression by increasing binding of the transcription factors HNF-1 and Sp1 to the SGLT1 gene (*Slc5a1*), and the transcript stabilizer HuR to SGLT1 mRNA. SGLT1 may contribute to cardiac injury in obesity and insulin resistance by stimulating ROS through its interaction with EGFR. SGLT1 may represent a therapeutic target for inhibition to prevent or to reverse diabetic cardiomyopathy.

**Supplementary Information:**

The online version contains supplementary material available at 10.1007/s00395-025-01136-7.

## Background

Diabetes mellitus can lead to a cardiomyopathy independent of other risk factors such as coronary artery disease and hypertension. There is an independent association between diabetes and cardiac hypertrophy, accompanied by systolic and diastolic dysfunction in 24% and 40–75% of patients respectively [9]. The overall prevalence of diabetic cardiomyopathy in the population is 1.1% [12]. After other insults such as myocardial ischemia, type 2 diabetics are more susceptible to oxidative stress, impaired cardioprotection [17], and heart failure [37]. Despite its clinical importance, the mechanisms underlying diabetic cardiomyopathy are complex and incompletely understood [9, 18]. Hyperglycemia, hyperinsulinemia, increased circulating fatty acids and triacylglycerols, and increased inflammatory cytokines in diabetes all promote cardiomyocyte dysfunction, injury, and death. Intermediate pathways include advanced glycation end-products (AGEs), fibrosis, inflammation, apoptotic and necrotic cell death, the renin–angiotensin–aldosterone system, impaired Ca^2+^ handling, increased fatty acid utilization, lipotoxicity, mitochondrial dysfunction, altered insulin signaling, oxidative stress, impaired autophagy, and endoplasmic reticulum (ER) stress. Impaired coronary blood flow may also contribute to heart failure in diabetes [16].

There are two families of glucose transporters, the facilitative (GLUT) and the sodium-dependent (SGLT) transporters. The SGLTs function by secondary active transport using the Na^+^ gradient established by the Na^+^/K^+^-ATPase pump. Each SGLT isoform is encoded by a separate gene. SGLT1 is present in small intestinal enterocytes and renal proximal tubule cells. Although GLUT1 and GLUT4 were thought to be responsible for cardiomyocyte glucose uptake [40], we were the first to show that SGLT1 protein is expressed in cardiomyocytes, and its expression is increased in diabetic cardiomyopathy, ischemia, and glycogen storage cardiomyopathy secondary to mutations in the gene encoding the γ2 subunit of AMP-activated protein kinase (AMPK) (*PRKAG2*) [4, 5]. We and others have shown that SGLT1 is the only SGLT isoform expressed in the heart, although a related protein with low glucose affinity sodium-myoinositol cotransporter-1 (SMIT1) is also expressed [44], and one report suggests that SGLT2 may be expressed [31].

Recently, we determined that SGLT1 is upregulated during cardiac ischemia/reperfusion (I/R) by activation of the transcription factors Sp1 and HNF-1 and the mRNA binding protein HuR, and that SGLT1 contributes to I/R injury by interacting with epidermal growth factor receptor (EGFR), which in turn increases protein kinase C (PKC), nicotinamide adenine dinucleotide phosphate oxidase 2 (Nox2) activity, and production of reactive oxygen species (ROS) [24]. Because hyperglycemia can activate HNF-1 [30, 45] and increased ROS production is associated with diabetic cardiomyopathy, we hypothesized that upregulation of SGLT1 in cardiomyocytes in type 2 diabetes contributes to the pathophysiology of diabetic cardiomyopathy by increasing oxidative stress through EGFR-PKC-Nox2 dependent mechanisms. In this study, we first established that cardiomyocyte-specific knockdown of SGLT1 attenuates cardiomyopathy in vivo in mice fed a high fat diet (HFD) and attenuates ROS production in vitro in cardiomyocytes exposed to high glucose and insulin. Next, we determined the regulation of cardiac SGLT1 in type 2 diabetes. Hyperglycemia and hyperinsulinemia upregulate SGLT1 by activating Sp1, HNF-1, and HuR. ERK increases phosphorylation of SGLT1, which causes its translocation to the sarcolemma. Finally, we determined that SGLT1 contributes cardiomyopathy by increasing Nox2 activity.

## Methods

### Human cardiac tissue

Samples of left ventricular cardiac tissue from structurally normal hearts were generously provided by Andreas M. Beyer, PhD, at the Medical College of Wisconsin.

### Mouse models

All studies involving mice conformed to the *Guide for the Care and Use of Laboratory Animals* (NIH Publication No. 85–23, revised 1996) and were approved by the University of Iowa Institutional Animal Care and Use Committee. FVB/N strain transgenic mice with cardiomyocyte-specific RNA interference knockdown of SGLT1 (TG^SGLT1-DOWN^) were constructed as we have previously reported [24, 36]. Equal proportions of male and female TG^SGLT1-DOWN^ 8-week-old mice (20–25 g body mass) and wildtype littermates were used for all studies. Mice were fed a high fat diet (HFD, 60% kcal from fat; Research Diets, catalog # D12492) to establish a model of obesity and insulin resistance that recapitulates features of type 2 diabetes or were fed control chow (4% kcal from fat) for 20 weeks beginning at age 8 weeks. For terminal studies, mice were euthanized using CO_2_ inhalation and cervical dislocation.

### Blood glucose assay

After a 4-h fast, blood samples were obtained from a tail-tip stump. Blood glucose was quantified using a commercially available kit (Contour Next Blood Glucose Monitoring System).

### Echocardiography

As previously described [35], mice were sedated with midazolam (5 mg/kg subcutaneously). Cardiac function was measured at baseline and at different time points by echocardiography in Lightly sedated mice using a Vevo 2100 machine equipped with a 30-MHz probe. Images were acquired in the 2-D mode from short axis at the level of papillary muscles and in the parasternal long axis at a frame rate of 300 fps. Studies were performed at heart rates greater than 500 bpm. The heart rates were similar and not statistically different among the groups: WT-control 606 ± 30, TG^SGLT1-DOWN^-control 624 ± 47, WT-HFD 640 ± 48, and TG^SGLT1-DOWN^-HFD 590 ± 47 bpm.

### Ex vivo isolated perfused heart studies

Mice were euthanized using CO_2_ inhalation and cervical dislocation. Death was confirmed by ascertaining cardiac and respiratory arrest. Following euthanasia of mice, hearts were rapidly excised and perfused with oxygenated Krebs–Henseleit solution (NaCl 118.0 mM, NaHCO_3_ 25.0 mM, KCl 4.7 mM, KHPO_4_ 1.21 mM, MgSO_4__7H_2_O 1.22 mM, Glucose 11.0 mM, CaCl_2__H_2_O 1.84 mM) in a Langendorff isolated perfused heart system. The hearts were perfused at constant pressure at 80 mm Hg using peristaltic roller pumps. After at least 10 min of equilibration, monitoring of left ventricular (LV) pressure was initiated by balloon insertion. LV balloon volume was 30 μL. A water bath was used to maintain the temperature of the perfusion buffer at 37 °C. Hearts had an intrinsic heart rate of approximately 360 bpm. Hearts with a low intrinsic heart rate (< 300/min) in ex vivo Langendorff isolated perfused heart preparations were excluded from the study.

### Isolation of adult mouse ventricular cardiomyocytes

Mice were euthanized and hearts harvested and mounted on a Langendorff isolated perfused heart system as described above. Hearts were initially perfused with perfusion buffer (113 mM NaCl, 4.7 mM KCl, 0.6 mM KH_2_PO_4_, 0.6 mM Na_2_HPO_4_, 1.2 mM MgSO4, 10 mM Na-HEPES, 12 mM NaHCO_3_, 10 mM KHCO_3_, 0.032 mM phenol red, 30 mM taurine, 10 mM BDM, and 5.5 mM glucose, adjusted to pH 7.0). Hearts were subsequently digested with perfusion buffer supplemented with 50 μM CaCl_2_ and 300 U/ml collagenase II (Sigma). After perfusion, the heart was removed, placed in plating medium (89% MEM [minimum essential medium, Sigma 51412C], 1% ITS [liquid media supplement, Sigma I3146], 100 U/ml penicillin, 1 mM L glutamine, 4.48 mM NaHCO_3_, 1 mM HEPES, 0.2% BSA, and 5% FBS), and mechanically disrupted by forceps. With a sterile transfer pipette, the tissue was aspirated up and down 10 times to disperse the cells and tissue fragments. The suspension was then filtered through a nylon mesh filter, rinsed, and plated in culture medium (94% MEM, 1% ITS, 100 U/ml penicillin, 1 mM L glutamine, 4.48 mM NaHCO_3_, 1 mM HEPES, and 0.2% BSA). All cells were maintained at 37 °C in a 2% CO_2_ atmosphere.

### In vitro exposure of cardiomyocytes to high glucose or insulin

To simulate hyperglycemia, isolated adult mouse cardiomyocytes were incubated for 10 min, 30 min, 1 h, 6 h, or 24 h in the presence of up to 25 mM D-glucose or mannitol to maintain equal osmolality. To simulate hyperinsulinemia, cardiomyocytes were incubated for 10 min, 30 min, 6 h, or 24 h in the presence of 10 µg/ml insulin. Cell viability was assessed by trypan blue exclusion.

### DNA and RNA analyses

DNA and RNA extraction and PCR were performed as described [24, 36]. Primers for PCR are listed in Table 1.

**Table 1:** Polymerase chain reaction primers used

Target	Primer Sequence
*Nppb* Forward	5’-AGGCGAGACAAGGGAGAACA-3’
*Nppb* Reverse	5’-GGAGATCCATGCCGCAGA-3’
*Slc5a1* Exon 1 Forward	5’-ACTGCACACCTAGGAGCTG-3’
*Slc5a1* Exon 2 Reverse	5’-GTTCCATTCAAAGCCACCCA-3’
*Slc5a1* Exon 4 Forward	5’-GTTTTGGTGGTTGTGCTGGG-3’
*Slc5a1* Exon 6 Reverse	5’-CAGTGATTGCTAGCAGGATGA-3’
*Slc5a1* Exon 9 Forward	5’-CTCTCGGCCAAGAACATGTC-3’
*Slc5a1* Exons 10–11 Reverse	5’-AAGAGAGTACTGGCGCTGTT-3’
*Slc5a1* Exon 14 Forward	5’-CTGTACCGTTTGTGTTGGAGT-3’
*Slc5a1* Exon 15 Reverse	5’-TCAGACAACACAGGGCTTCT-3’

### Protein analyses

Protein was extracted from whole cardiac tissue or cell homogenates and analyses performed as we have described [35, 36]. In brief, to prepare whole tissue homogenates, heart tissue was homogenized in RIPA buffer with a Dounce homogenizer followed by centrifugation at 12,000 g for 30 min and collection of the supernatant containing the tissue homogenate. The membrane fraction was prepared from cardiac tissue and cell samples with a plasma membrane protein extraction kit (Abcam, ab65400) according to the manufacturer’s instructions. Nuclear extracts were prepared as described [39]. Prior to immunoblotting for phosphorylated SGLT1, samples were immunoprecipitated by incubation overnight with anti-SGLT1 primary antibody at 4 °C, and then conjugated and pulled down with protein A agarose beads (Pierce). An anti-phospho-serine/threonine antibody was used to detect phosphorylated SGLT1.

The following primary antibodies were used: ERK (Cell Signaling, 9102, RRID: AB_330744), HNF-1 (Cell Signaling, 89,670, RRID: AB_2728751), HuR (Abcam, ab136542), lamin B (Cell Signaling, 13,435, RRID: AB_2737428), phospho-ERK (Cell Signaling, 9101, RRID: AB_331646), ERK (Cell Signaling, 9102, RRID:AB_330744), Na^+^/K^+^-ATPase (Cell Signaling, 3010, RRID:AB_2060983), PKC (Sigma-Aldrich, P5704, RRID: AB_477375), PKC β (BD Biosciences, 51–9,002,039), phospho-PKC (pan) (Cell Signaling, 9379, RRID: AB_2252807), SGLT1 (Abcam, ab14686, RRID: AB_301411), SMIT1 (Assay Biotech, C18843, RRID:AB_10684976), Sp1 (Abcam, ab13370, RRID: AB_300283), phospho-Sp1 (Abcam, ab59257, RRID: AB_946335), and α-tubulin (Cell Signaling, 11H10). The following secondary antibodies were used: anti-rabbit (Cell Signaling, 7074, RRID: AB_2099233) and anti-mouse (Cell Signaling, 7076, RRID: AB_330924).

### Co-immunoprecipitation assays

Co-immunoprecipitations assays were performed to determine the interaction between SGLT1, ERK, and Sp1. Cardiac tissue was homogenized in lysis buffer (20 mM Tris HCl, pH 8, 137 mM NaCl, 1% Nonidet P-40 [NP-40], 2 mM EDTA) at 4 °C, the homogenate was centrifuged, and the supernatant collected. 10–50 μg cell lysate was incubated with primary antibody at 4 °C for 1 h, following which 100 μL protein A agarose beads were added at 4 °C for 4 h. The samples were centrifuged, and the pellets washed 3 times with lysis buffer at 4 °C. The antigen–antibody complex was eluted from the beads by heating the samples in loading buffer (2 × Laemmli Sample Buffer, Bio-Rad) with denaturant SDS without DTT at 50 °C for 10 min, and centrifuging. The supernatant was transferred to a new tube with 100 mM DTT (elution 1). The pellets were resuspended in SDS buffer with DTT (elution 2). The eluted samples were boiled for 5 min and analyzed by immunoblot.

### Histopathology

Sections were stained with Masson’s trichrome to assess interstitial fibrosis [35]. For quantitative analyses of staining, five separate regions per slide were photographed at 40X. Micrographs were digitized and analyzed using Image J 1.44p software (National Institutes of Health, USA) in a blinded fashion.

### Immunohistochemistry studies

Paraffin-embedded heart tissue sections were first deparaffinized and then incubated with Tris/EDTA pH 9.0 buffer at 95 °C for 15 min for antigen retrieval. The slides were then incubated with respective primary antibodies at 4 °C overnight, followed by application of streptavidin peroxidase and incubation for 10 min at room temperature. The slides were rinsed four times in buffer. Thirty μL of 3'-diaminobenzidine (DAB) chromogen mixed with 1.5 ml of DAB substrate were applied to the tissue specimens and incubated for 1–10 min. Following four rinses in buffer, hematoxylin was added as a counterstain. The following primary antibodies were used: collagen I (Invitrogen, PA1-26,204, RRID:AB_2260734) and Nox2 (Abcam, ab80897, RRID:AB_1640390).

### Chromatin immunoprecipitation (ChIP) and RNA immunoprecipitation (RIP)

ChIP was performed to quantify the binding of the transcription factors specificity protein 1 (Sp1) and hepatocyte nuclear factor 1 (HNF-1) to the SGLT1 gene (*Slc5a1*) promoter, and RIP to quantify binding of HuR to a uridine-rich element in the 3’ untranslated region (3’ UTR) of the SGLT1 mRNA. ChIP analyses were performed as we have described [5] with Sp1 and HNF-1 primary antibodies using a commercially available kit (Cell Signaling, 9002). RIP analyses were performed as described [24] with HuR primary antibody followed by QPCR to quantify HuR bound SGLT1 mRNA.

### SGLT1 cDNA transfection

Cardiomyocytes were plated in six-well plates until 70–90% confluent. Fifteen µl Lipofectamine 2000 (ThermoFisher, 11,668,019) was diluted in 150 µl opti-MEM reduced serum medium (ThermoFisher, 31,985,070). Plasmid cDNA-lipid complexes were prepared by mixing diluted Lipofectamine and 14 µg cDNA (SinoBiological, HG15230-ACG). One hundred fifty µL cDNA-lipid complexes were added to the cells. Cells were incubated at 37 ºC for 24 h.

### Protein kinase C (PKC) activity

PKC activity was measured with a commercially available kit (Abcam, Ab139437) following the manufacturer’s instructions.

### Superoxide production

To monitor superoxide production in live cardiomyocytes directly in real time, a commercially available kit (Abcam, Ab139477) was used. Upon staining of the cardiomyocytes, the fluorescent product generated was visualized using a wide-field fluorescence microscope equipped with standard red (650/670 nm) fluorescent cubes.

### Malondialdehyde (MDA) production

Differences in reactive oxygen species generation were estimated by measurement of MDA production using a commercially available kit (Abcam, Ab118970) following the manufacturer’s instructions.

### Statistical analysis

Data are presented as mean ± standard error (SE). Data were assessed for normality with the Shapiro–Wilk normality test. Comparisons between two groups were made using the Student’s *t*-test. For comparisons among three or more groups, one-way ANOVA was used, followed by post hoc Bonferroni correction. A *P*-value less than 0.05 was considered significant.

## Results

### Cardiac SGLT1 expression is upregulated in a model of obesity and insulin resistance

**Fig. 1:**
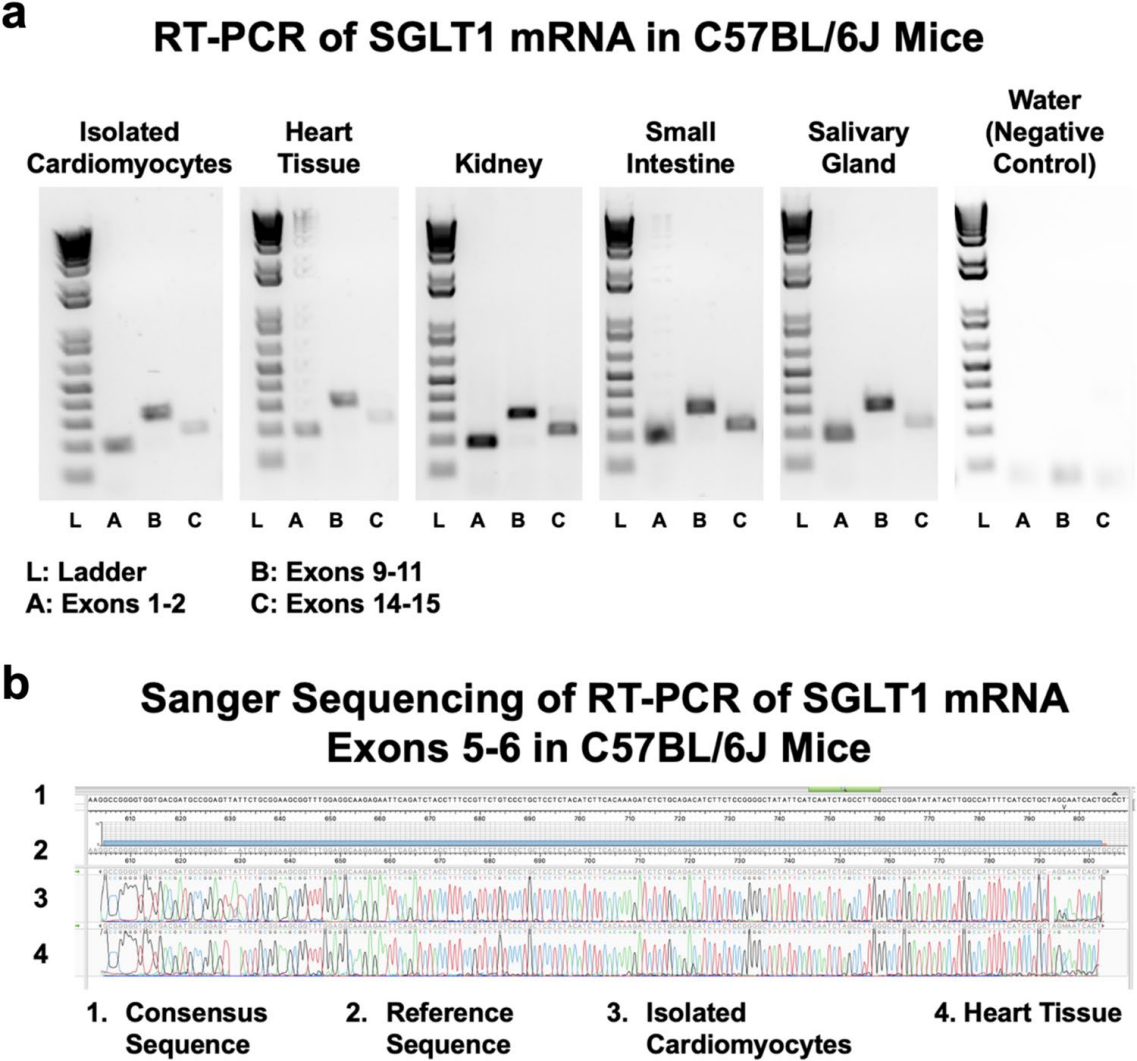
Full-length SGLT1 Is expressed in cardiomyocytes. **a** RT-PCR of SGLT1 mRNA from isolated cardiomyocytes and whole tissue from C57BL/6 J mice using primers to amplify the 5’ end (lane **A**, exons 1–2), the middle (lane **B**, exons 9–11) and the 3’ end (lane **C**, exons 14–15) of the transcript. Full length SGLT1 mRNA comprising all 15 exons was present in all specimens, including isolated cardiomyocytes and total cardiac tissue. The ladder (1 Kb Plus DNA Ladder, Invitrogen) sizes 100–15,000 bp double-stranded DNA. **b** RT-PCR products for exons 5–6 were subjected to Sanger sequencing to confirm the specificity of the amplification

**Fig. 2:**
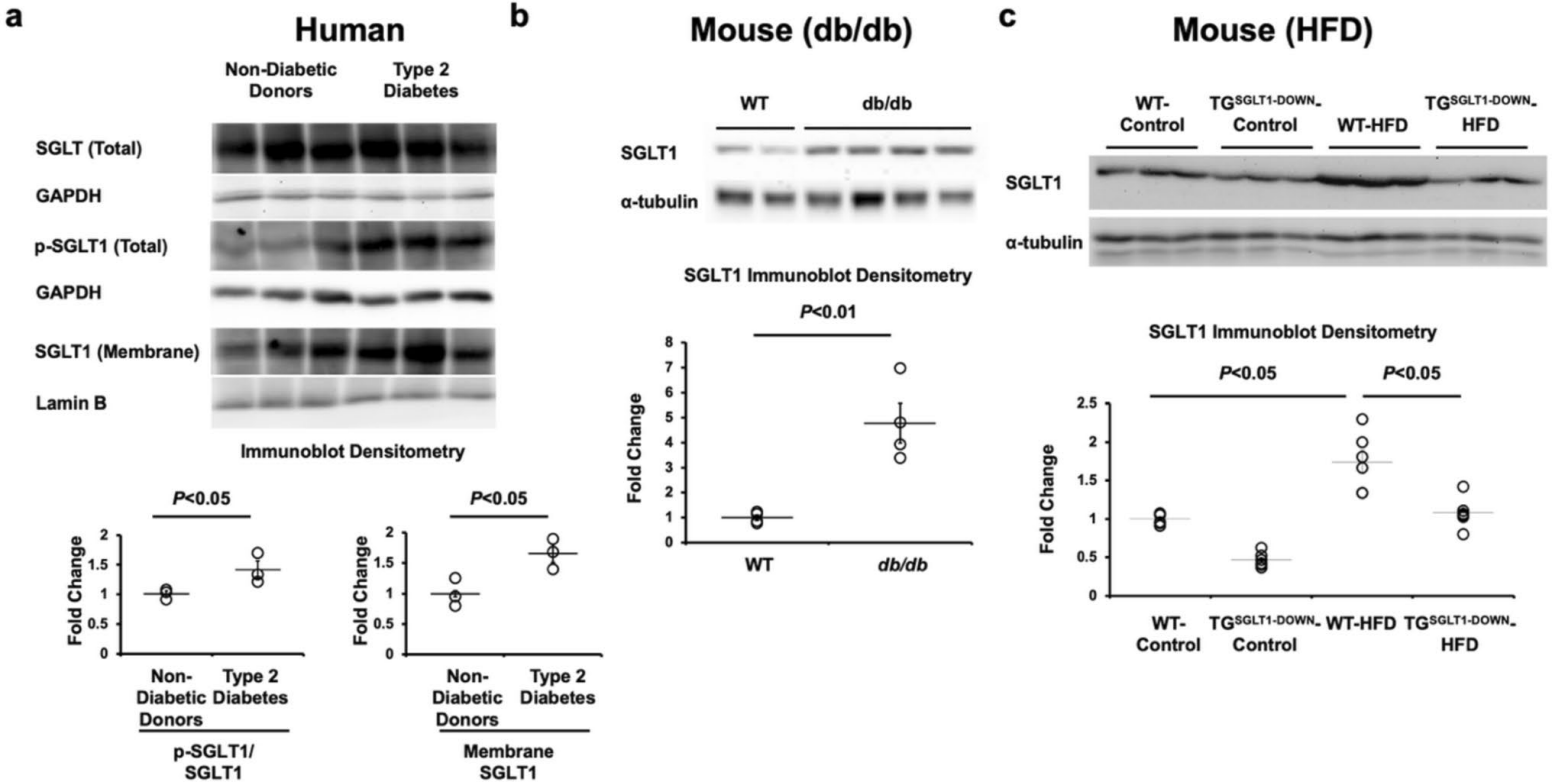
Cardiac SGLT1 expression is upregulated in diabetes. Cardiac membrane SGLT1 and total phosphorylated (p-SGLT1) protein was upregulated in **a** human subjects with type 2 diabetes (*n* = 3/group) and **b** db/db (*n* = 4/group) and **c** high fat diet (HFD) (n = 3/group) mouse models of type 2 diabetes. Eight-week-old WT and TG^SGLT1-DOWN^ mice were fed a HFD (60% kcal from fat) or control chow (4% kcal from fat) for 20 weeks (*n* = 5/group). The upregulation of SGLT1 was attenuated in TG^SGLT1-DOWN^ mice fed a HFD. α-tubulin or lamin B were used as loading controls. Each sample shown is a separate biological replicate representing an individual mouse or human

RT-PCR showed that full-length SGLT1 mRNA, comprising all 15 exons, was expressed in isolated cardiomyocytes and total cardiac tissue from wildtype (WT) mice, as well as in other tissues (Fig. 1A). The specificity of the PCR amplification was confirmed by Sanger sequencing (Fig. 1B). We previously reported that cardiac SGLT1 mRNA was increased approximately 200% in an initial cohort of human subjects with diabetic cardiomyopathy and in *ob/ob* type 2 diabetic mice [4]. In the current study, we extended these findings in left ventricular (LV) free wall tissue to a second cohort of human subjects with type 2 diabetes and without coronary artery disease, hypertension, or valve disease (contrasted with nondiabetic unmatched heart donors) (Table 2), and in *db/db* type 2 diabetic mice. Phosphorylation of SGLT1 causes its translocation to the sarcolemma. Phosphorylated and membrane-bound cardiac SGLT1 protein were increased approximately 50% in human subjects with type 2 diabetes (Fig. 2A), and total cardiac SGLT1 protein was increased approximately 400% in *db/db* mice at age 24 weeks (Fig. 2B) and approximately 100% in WT FVB/N mice fed a high fat diet (HFD) for 20 weeks beginning at age 8 weeks (Fig. 2C). This upregulation of SGLT1 was attenuated in TG^SGLT1-DOWN^ mice fed a HFD.

**Table 2:** Sources of human cardiac tissue

Sample	Age	Sex	Race	Diabetes
1	58	Male	White	No
2	49	Male	White	No
3	22	Female	White	No
4	58	Male	Black	Type 2
5	67	Female	White	Type 2
6	52	Female	White	Type 2

### Transgenic knockdown of cardiomyocyte SGLT1 Attenuates cardiomyopathy in vivo

**Fig. 3:**
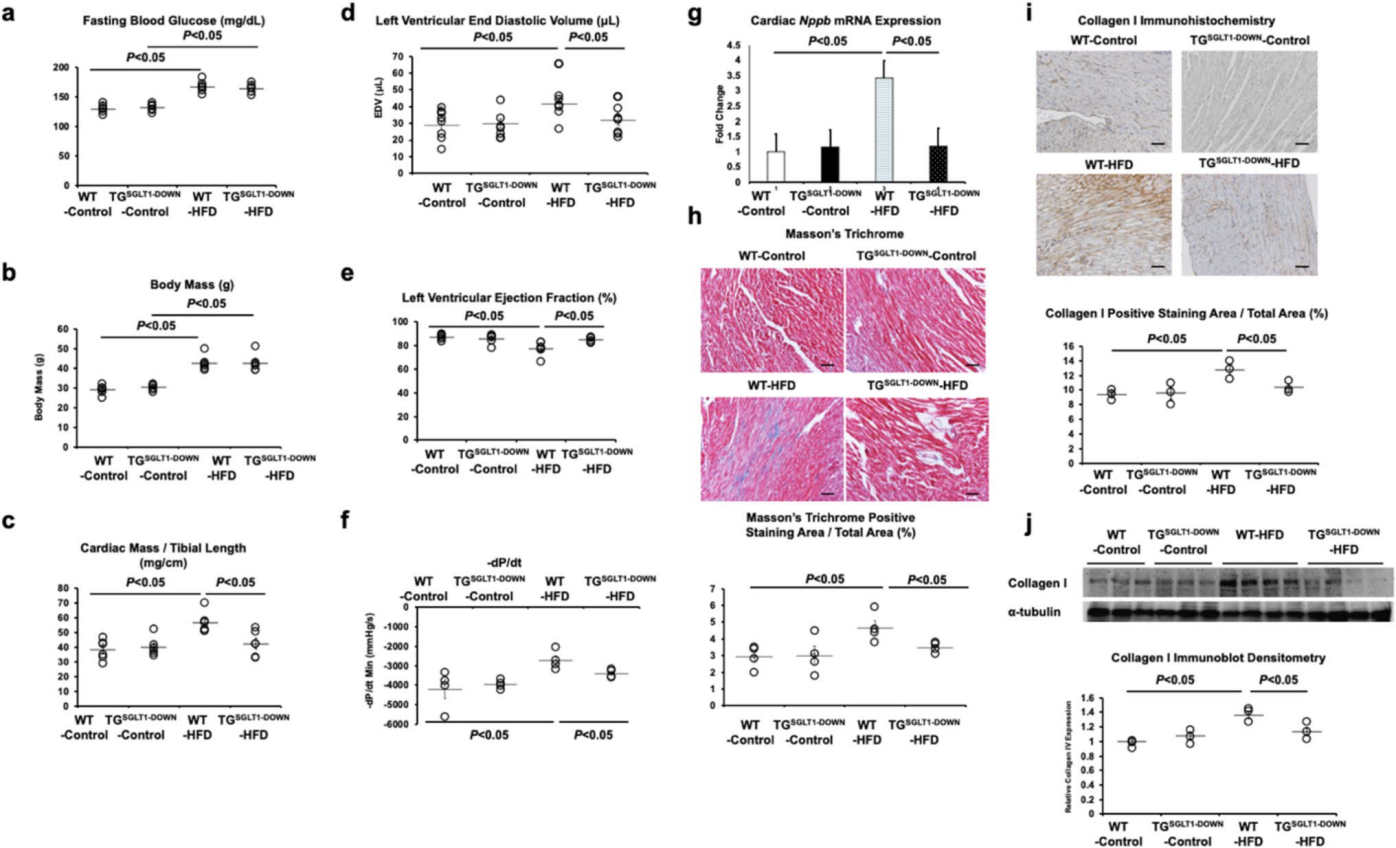
Transgenic knockdown of cardiomyocyte SGLT1 attenuates cardiomyopathy in vivo. Eight-week-old TG^SGLT1-DOWN^ mice and WT Littermates were fed a HFD or control chow for 20 weeks (*n* = 4–6/group, with equal numbers of males and females, as shown in the figures). HFD caused similar increases in **a** fasting blood glucose and **b** body mass in WT and TG^SGLT1-DOWN^ mice. No significant sex differences were observed. Relative to WT mice exposed to HFD, TG^SGLT1-DOWN^ mice were protected against **c** cardiac hypertrophy as assessed by the ratio of cardiac mass to tibial length after sacrifice, **d** left ventricular dilation and **e** deterioration in left ventricular ejection fraction (LVEF) on echocardiography before sacrifice, and **f** deterioration of diastolic function on ex vivo hemodynamic studies. TG^SGLT1-DOWN^ mice had lower cardiac expression of *Nppb* on QPCR **g**, less cardiac fibrosis on Masson trichrome staining **h**, and less cardiac expression of collagen I on immunohistochemistry **i** and immunoblot **j**. Scale bars on histology images represent 100 μm. α-tubulin was used as a loading control on the immunoblot

TG^SGLT1-DOWN^ mice and WT Littermates were fed HFD or control chow for 20 weeks beginning at age 8 weeks. WT-HFD and TG^SGLT1-DOWN^-HFD mice had similar increases in fasting plasma glucose and body mass (Fig. 3A, B) that are typical for this murine model of obesity and insulin resistance [46]. WT-HFD mice exhibited a cardiomyopathy phenotype consistent with prior reports [19, 27, 38, 41], characterized by cardiac hypertrophy (ratio of cardiac to tibial length) on necropsy at age 28 weeks (Fig. 3C), LV dilation (Fig. 3D) and impairment of LV ejection fraction (LVEF) on in vivo echocardiography (Fig. 3E), impairment of diastolic function as assessed by –dP/dt on ex vivo hemodynamic studies (Fig. 3F), increased cardiac expression of *Nppb* (Fig. 3G), fibrosis on Masson trichrome staining (Fig. 3H), and increased expression of collagen I protein on immunohistochemistry (Fig. 3I) and immunoblot (Fig. 3J). Relative to WT-HFD mice, all these features of cardiomyopathy were attenuated or abrogated in TG^SGLT1-DOWN^-HFD mice. However, on ex vivo hemodynamic studies, TG^SGLT1-DOWN^-HFD mice were not protected against impaired + dP/dt and LV developed pressure was not significantly different among the four groups (Online Resource Supplementary Fig. 1). The LV end-diastolic pressures were nearly 0 mmHg in all groups. (Fig. 3 is reproduced in the Online Resource Supplementary Fig. 2, in which versions of the graphs with truncated ordinates are depicted to allow the individual data symbols to be more easily distinguished.)

### Potential regulatory mechanisms of cardiomyocyte SGLT1 in diabetes

In our previous studies of cardiac ischemia [24], we demonstrated that SGLT1 expression is regulated by several mechanisms. Increased binding of the transcription factors HNF-1 and Sp1 to the promoter of the SGLT1 gene (*Slc5a1*) increases expression of the gene. There is increased binding of the protein HuR to the 3’ untranslated region (3’ UTR) in the SGLT1 mRNA, which is known to increase its stability [28]. Activation of ERK leads to increased phosphorylation of Sp1, HuR and the HNF-1 co-factor Mirk which may be responsible for increased SGLT1 expression at both the transcript and the protein level. ERK increases phosphorylation of SGLT1, which causes its translocation to the sarcolemma.

**Fig. 4:**
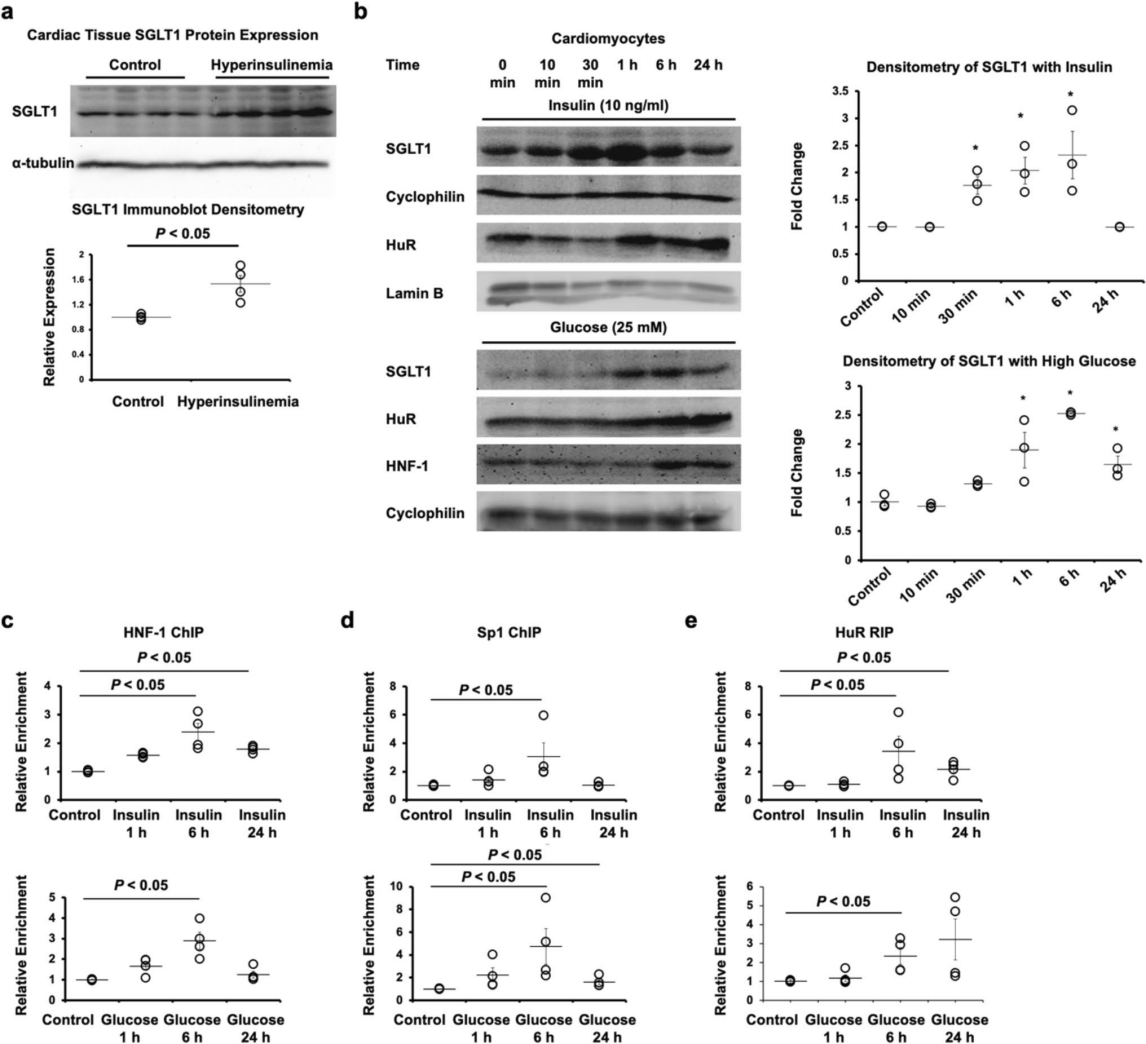
Transcriptional regulatory mechanisms of cardiomyocyte SGLT1 following exposure to high glucose and insulin. **a** WT mice exposed to a hyperinsulinemic-euglycemic clamp for 1 h showed an increase in cardiac sarcolemma-bound SGLT1 expression (*n* = 4/group). **b** In cardiomyocytes exposed to insulin (10 µg/ml) or high glucose (25 mM), there was a time-dependent increase in SGLT1 expression that was associated with an increase in HuR and/or HNF-1 expression. Densitometry of 3 blots is shown. *, *P* < 0.05 compared to respective control (*n* = 3/group). **c**–**e** Exposure of cardiomyocytes cells to insulin and glucose increased binding of Sp1 and HNF-1 to the SGLT1 gene promoter as assayed by chromatin immunoprecipitation (ChIP) and HuR to SGLT1 transcript assayed by RNA immunoprecipitation (RIP). *, *P* < 0.05 compared to respective control (*n* = 4/group). α-tubulin, cyclophilin, or lamin B were used as loading controls on the immunoblots. Representative immunoblots are shown

In the current study, we further interrogated the mechanism of SGLT1 upregulation in diabetes. Cardiac SGLT1 protein was increased in vivo in mice exposed to a hyperinsulinemic-euglycemic clamp (Fig. 4A). This finding is consistent with our earlier demonstration that insulin-stimulated cardiac glucose uptake in vivo is sensitive to SGLT1 inhibition [4]. Exposure of isolated cardiomyocytes in vitro to high glucose or insulin increased SGLT1 protein expression that was maximal at approximately 1 h and was associated with an increase in HuR and/or HNF-1 expression (Fig. 4B). Chromatin immunoprecipitation (ChIP) assays showed that high glucose and insulin increased binding of the transcription factors HNF-1 and Sp1 to the *Slc5a1* promoter, thereby increasing transcription of SGLT1 (Fig. 4C, D). RNA immunoprecipitation (RIP) assays showed that high glucose and insulin increased binding of HuR to the SGLT1 mRNA (Fig. 4E). These findings suggest that increased transcription of *Slc5a1* and increased stability of the transcript underlie the increased expression of SGLT1.

**Fig. 5:**
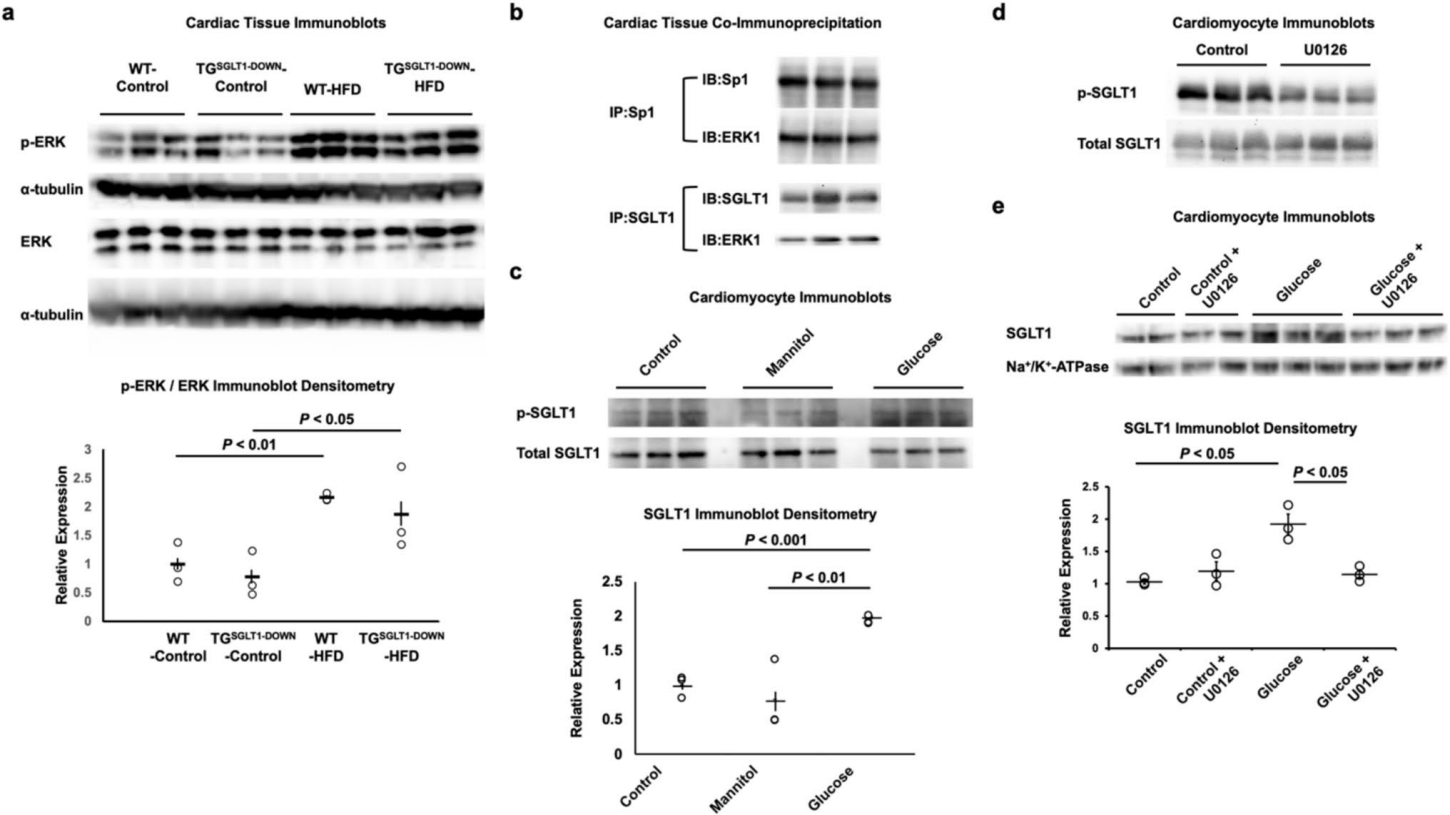
ERK regulation of cardiomyocyte SGLT1 in diabetes. **a** Phosphorylated ERK (p-ERK) expression in cardiac tissue from WT and TG.^SGLT1-DOWN^ mice fed a control diet or HFD. α-tubulin was used as a loading control on the immunoblot (*n* = 3/group). **b** Total cardiac protein from wildtype mice was immunoprecipitated by anti-Sp1 or anti-SGLT1 primary antibody and the immunoprecipitate probed on immunoblots for ERK1. ERK1 directly interacts with SGLT1 and Sp1 (*n* = 3/group). **c** SGLT1 phosphorylation in cardiomyocytes exposed to control (physiological) glucose, mannitol (25 mM), or high glucose (25 mM) (*n* = 3/group). **d** SGLT1 phosphorylation was decreased in cardiomyocytes exposed to the ERK inhibitor U0126 (*n* = 3/group). **e** SGLT1 expression in the sarcolemma of cardiomyocytes exposed to control (physiological) or high glucose in the absence or presence U0126 (1 μM for 24 h). Representative immunoblots are shown (*n* = 3/group)

The phosphorylated, active form of ERK was upregulated following HFD in both WT and TG^SGLT1-DOWN^ hearts (Fig. 5A). Co-immunoprecipitation (co-IP) studies suggested that ERK1 interacts with both Sp1 and SGLT1 (Fig. 5B). The phosphorylation of SGLT1 was increased in isolated cardiomyocytes exposed to high glucose in vitro (Fig. 5C), whereas the ERK inhibitor U0126 attenuated phosphorylation of SGLT1 (Fig. 5D). Furthermore, the phosphorylation of SGLT1 in the presence of high glucose was associated with increased expression in the sarcolemma, an effect that was abrogated by U0126 (Fig. 5E). Collectively, these data suggest that ERK directly phosphorylates SGLT1 (which is known to cause its translocation to the sarcolemma) and Sp1 (which is known to increase its binding to the *Slc5a1* promoter and its consequent transcription).

### SGLT1 contributes to cardiac oxidative stress in a model of obesity and insulin resistance

**Fig. 6:**
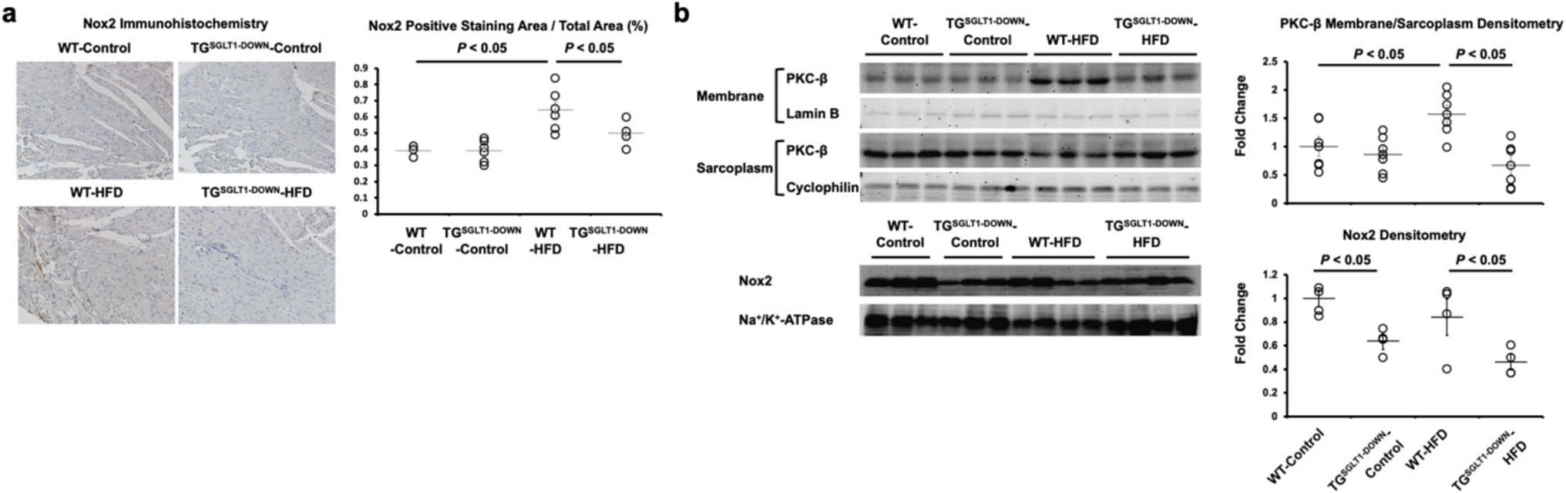
SGLT1 mediates PKC-Nox2 pathway in diabetic cardiomyopathy. Relative to WT mice, TG^SGLT1-DOWN^ mice exposed to HFD exhibited less cardiac expression of Nox2 **a**, that was associated with less expression of active, membrane-associated PKC-β **b** (*n* = 4–7/group)

**Fig. 7:**
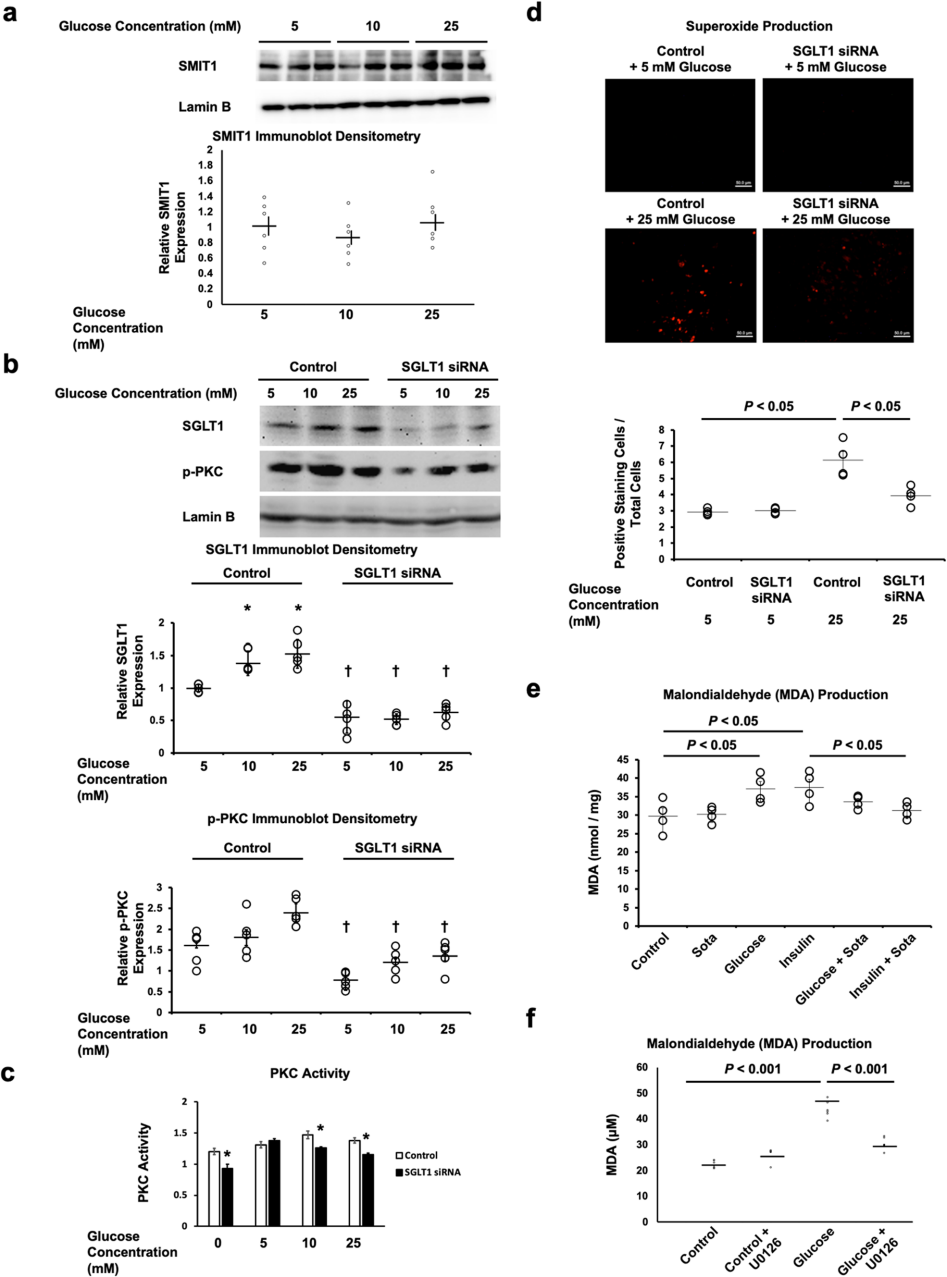
SGLT1 mediates oxidative stress in the presence of high glucose and insulin. Cardiomyocytes were exposed to SGLT1 siRNA (knockdown) or scrambled siRNA (control) for 24 h, followed by exposure to glucose at concentrations 5–25 mM in the culture medium for 24 h. **a** There was no association between glucose concentration and SMIT1 expression. **b** In contrast, there was a glucose concentration-dependent increase in SGLT1 expression that was attenuated with SGLT1 knockdown. When SGLT1 was knocked down by siRNA, there was **b** decreased expression of phosphorylated, active PKC (p-PKC) at all glucose concentrations (*, *P* < 0.05 relative to control at glucose 5 mM; ✝, *P* < 0.05 relative to control cells at the equivalent glucose concentration) (*n* = 5/group), **c** decreased PKC activity as assayed by a commercially available colorimetric kit (*, *P* < 0.05 relative to control cells at the equivalent glucose concentration), and **d** decreased superoxide production as assayed by a commercially available fluorescent kit (*n* = 4/group). **e** Pharmacological inhibition of SGLT1 by sotagliflozin (sota, 100 nM for 24 h) abrogated the production of malondialdehyde (MDA), a marker of lipid peroxidation, in response to high glucose or insulin (*n* = 4/group). **f** The ERK inhibitor U0126 also abrogated the production of MDA in response to high glucose

**Fig. 8:**
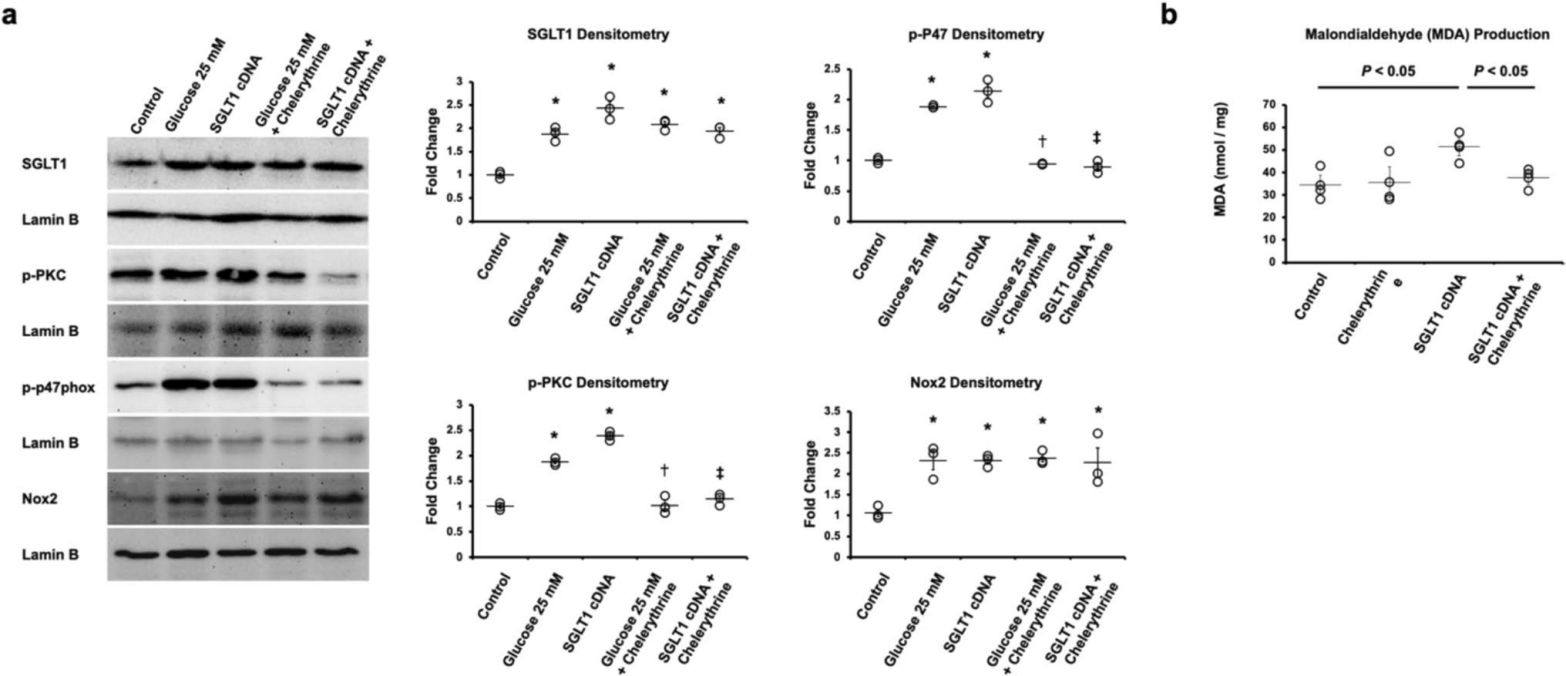
PKC mediates the effects of SGLT1 on oxidative stress. **a** Expression of SGLT1, p-PKC, p-p47 and Nox2 in cardiomyocytes after exposure to high glucose or SGLT1 cDNA transfection, with and without the PKC inhibitor chelerythrine. Densitometry reflects three separate experiments. *, *P* < 0.05 compared to control; ✝, *P* < 0.05 compared to glucose 25 mM; ‡, *P* < 0.05 compared to SGLT1 cDNA (*n* = 3/group). **b** Production of malondialdehyde (MDA), a marker of oxidative stress, following SGLT1 cDNA transfection with and without chelerythrine (*n* = 4/group)

We have previously reported [24] that SGLT1 binds to the autophosphorylation domain of EGFR, which in turn activates PKC. Subsequently, p47phox, a subunit of Nox2, is phosphorylated, leading to oxidative stress. PKC isoforms also are known to induce insulin resistance and lower NO production [18]. We determined whether these pathways were active in the setting of obesity and insulin resistance. In vivo, TG^SGLT1-DOWN^ hearts had decreased expression of PKC and Nox2 relative to WT hearts following HFD (Fig. 6A, B). In vitro, exposure of isolated cardiomyocytes to increasing glucose concentrations (5–25 mM) had no effect on the expression of SMIT1 in the presence of increasing concentrations of glucose (Fig. 7A). In contrast, increasing concentrations of glucose were associated with increasing expression of SGLT1 in cardiomyocytes (Fig. 7B), and increased superoxide production (Fig. 7D). High glucose was not associated with increases in the activated, phosphorylated form of PKC (Fig. 7B) or in PKC activity (Fig. 7C). However, SGLT1 siRNA knockdown decreased phosphorylated PKC (Fig. 7B) and PKC activity (Fig. 7C) from baseline at all glucose concentrations. Furthermore, SGLT1 siRNA knockdown decreased superoxide production associated with high glucose (Fig. 7D). Similarly, pharmacological inhibition of SGLT1 by sotagliflozin abrogated the production of malondialdehyde (MDA), a marker of lipid peroxidation, in response to high glucose or insulin (Fig. 7E). Like SGLT1 knockdown, the ERK inhibitor U0126, which decreases SGLT1 phosphorylation and sarcolemma expression (Fig. 5D, E), also decreased MDA production (Fig. 7F). Exposure to either high glucose or SGLT1 transfection in cardiomyocytes were both associated with PKC and p47phox phosphorylation, that was abrogated by PKC inhibition with chelerythrine (2.5 µM) (Fig. 8A). Similarly, MDA production in response to SGLT1 cDNA transfection was attenuated by chelerythrine (Fig. 8B). Collectively, these data suggest that, in the presence of high glucose or insulin, SGLT1 leads to ROS production.

## Discussion

Our studies confirm that cardiac SGLT1 is upregulated both in human subjects with type 2 diabetes and diabetic cardiomyopathy and in murine models of diabetic cardiomyopathy. Although Beauloye and colleagues recently suggested that cardiomyocytes express a truncated form of SGLT1 that is not membrane-bound [14], we determined by RT-PCR that full-length SGLT1 mRNA is expressed in isolated cardiomyocytes and total cardiac tissue as well as in other tissues (Fig. 1). Other investigators recently reported the same finding [1]. In addition, we [4, 5, 24, 36], our collaborators [23] and other investigators [42] have previously shown by immunofluorescence and membrane fractionation studies that full-size SGLT1 protein is localized to the sarcolemma and that SGLT1 mediates Na^+^ and glucose uptake in cardiomyocytes. However, it is possible that a truncated SGLT1 is also present and plays a role in signaling pathways.

The upregulation of cardiac SGLT1 is initiated by hyperglycemia and hyperinsulinemia in type 2 diabetes (Fig. 4). Our studies have elucidated the specific regulatory mechanisms responsible for this upregulation. Insulin and IGF have been previously reported to activate ERK through a signaling cascade involving Grb2, SOS, and Raf [8]. ERK leads to upregulation of SGLT1 through multiple targets, including (1) phosphorylation of the transcription factor Sp1 and phosphorylation of the HNF-1 co-factor Mirk, which lead to increased transcription of *Slc5a1*; phosphorylation of HuR, which stabilizes the SGLT1 mRNA transcript; and phosphorylation of SGLT1, which causes its translocation to the sarcolemma. Consistent with these data, Raf has been previously reported to upregulate SGLT1 [33]. Hyperinsulinemia-mediated increases in SGLT1 expression are relevant in type 2 diabetes because, despite generalized insulin resistance, hyperinsulinemia is associated with ERK activation through the IGF-1 receptor [43].

Hyperglycemia also activates HNF-1, Sp1, and HuR, leading to increased expression of SGLT1. These findings are consistent with reports that Sp1 binding activity is increased by O-glycosylation [47], and that Sp1 expression is increased by glucose [21], insulin [3], and AGEs [7]. SGLT1 itself may also be activated by glycosylation [2]. Other investigators have now replicated our finding that cardiac SGLT1 expression is increased in a HFD-streptozotocin (STZ) mouse model of type 2 diabetes in vivo [42] and that high glucose increases SGLT1 expression in cardiomyocytes in vitro [11]. However, there have been divergent reports of decreased expression of SGLT1 in models of type 2 diabetes [1].

We have previously shown in cardiac ischemia/reperfusion injury that cardiac SGLT1 interacts with EGFR to activate PKC, which in turn activates Nox2 and leads to increased ROS-production [24]. We hypothesized that upregulation of cardiac SGLT1 in diabetes leads to a similar increase in oxidative stress through PKC. Although we did document increased PKC activity and Nox2 expression in vivo (Fig. 6), we did not consistently document an activation of PKC in the presence of high glucose in vitro (Fig. 7B, C). Therefore, it is possible that PKC activation in diabetes in vivo is mediated by a mechanism other than high glucose. However, despite baseline differences in the in vitro and the in vivo systems, SGLT1 knockdown did abrogate the increase in PKC activity and Nox2 expression in vivo and did decrease baseline PKC activity in vitro, associated with decreased ROS production. Oxidative stress ultimately leads not only to cardiomyocyte injury and dysfunction, but also extracellular cardiac fibrosis. Thus, we observed several features of diabetic cardiomyopathy, including cardiac hypertrophy and dilation, systolic and diastolic dysfunction, and fibrosis. In addition, activation of EGFR by SGLT1 may lead to increased ROS production by other means, because EGFR is known to drive mitochondrial ROS production [49]. RNAi-mediated or pharmacological inhibition of SGLT1 attenuates or abrogates these features. Other investigators have recently corroborated our findings that RNAi-mediated knockdown or pharmacological inhibition of SGLT1 decreases ROS [10, 11, 13], apoptosis [13, 26], and pyroptosis [10] in cardiomyocytes exposed to high glucose in vitro; and decreases ROS, inflammation, apoptosis, mitochondrial dysfunction, and LV dysfunction in HFD-STZ mice [42, 48] and rats [26] in vivo. Thus, activation of ROS appears to be at least one potential mechanism by which SGLT1 contributes to diabetic cardiomyopathy.

Reports that glucose fluctuation may accentuate SGLT1-mediated injury [10, 42, 48] are consistent with our finding that the upregulation of SGLT1 in cardiomyocytes exposed to high glucose in vitro appears to be biphasic, with a peak at 1–6 h (Fig. 4B). Intermittent hyperglycemia, as often seen in diabetic patients, may lead to a sustained upregulation of SGLT1. This biphasic expression of SGLT1 that is dependent on intermittent elevation of glucose may also underlie differences in SGLT1 expression observed in other models of diabetes. For example, we previously observed decreased cardiac expression of SGLT1 in STZ diabetic mice, a model of type 1 diabetes [4], and other investigators observed decreased cardiac expression of SGLT1 in Zucker Diabetic Fatty (ZDF) rats, a model of type 2 diabetes [1]. These models may have experienced sustained, rather than fluctuating, increases in circulating glucose. In addition, in the case of STZ diabetic mice, this model is characterized by hypoinsulinemia, which should be associated with decreased cardiomyocyte expression of SGLT1 (Fig. 4B).

Other investigators have suggested that SMIT1, rather than SGLT1, may mediate Nox activation and ROS production in cardiomyocytes exposed to high glucose [44]. They studied global SGLT1 knockout mice that required a glucose- and galactose-free diet to survive, which may have led to effects unrelated to cardiomyocyte-specific knockdown of SGLT1 as in our experiments. They also used analogs of glucose that had different specificities for different transporters. However, we do not discount the possibility that SMIT1 plays a role in addition to SGLT1.

Although our studies have focused on cardiomyocytes, SGLT1 may also contribute to the pathophysiology of diabetic cardiomyopathy in non-cardiomyocyte cells. RNAi-mediated knockdown of SGLT1 in cardiac fibroblasts exposed to high glucose decreased proliferation and migration of fibroblasts and collagen synthesis [25]. SGLT1 may also mediate endothelial cell senescence in the presence of high glucose [20, 34].

Another limitation of our study is that the attenuation of dysfunction observed TG^SGLT1-DOWN^-HFD mice in vivo (LVEF, Fig. 3E) was not replicated in our ex vivo hemodynamic studies (+ dP/dt and left ventricular developed pressure, Online Resource Supplementary Fig. 1). It is possible that the perfusate used in the Langendorff isolated perfused heart system does not reflect metabolic substrates used by the hearts in vivo, and that the systolic dysfunction in WT-HFD mice is dependent on the composition of the in vivo substrates. It is also possible that TG^SGLT1-DOWN^-HFD and WT-HFD mice have in vivo physiological differences, such as intravascular volume, loading conditions, or neurohormonal environment, that were absent ex vivo. However, we believe that such physiological differences are less likely because the TG^SGLT1-DOWN^ mice have cardiomyocyte-specific knockdown of SGLT1 and should have preserved SGLT1 expression elsewhere.

SGLT2 inhibitors have been shown to improve heart failure regardless of the presence of diabetes or the extent of blood glucose lowering [29, 32]. Our study suggests that additional SGLT1 inhibition may be a novel therapeutic strategy for diabetic cardiomyopathy and for heart failure more generally. Canagliflozin, a dual SGLT1/SGLT2 inhibitor, was recently shown to reduce Nox2 activity and ROS in human atrial myocardium and in human ventricular cardiomyocyte cell lines [22]. These effects were attributed to SGLT1 inhibition because SGLT2 was not expressed in the target tissue. Furthermore, a genetic variant associated with decreased SGLT1 function was shown in normal human subjects to improve systolic function on echocardiography [6]. These human studies provide further impetus for additional translational studies targeting SGLT1 in diabetic cardiomyopathy and other forms of heart failure.

## Conclusions

Hyperglycemia and hyperinsulinemia upregulate cardiomyocyte SGLT1 by activating ERK, the transcription factors HNF-1 and Sp1, and the transcript stabilizer HuR. SGLT1 mediates cardiac injury by stimulating the production ROS, possibly by activating PKC and Nox2. SGLT1 may represent a therapeutic target for inhibition to prevent or to reverse diabetic cardiomyopathy. SGLT1/SGLT2 dual inhibitors with greater SGLT2 specificity have been approved for clinical use and may provide evidence of the relative benefits of SGLT1 and SGLT2 inhibition. However, a complete understanding of the clinical benefits of SGLT1 inhibition will likely require the development and investigation of more specific SGLT1 inhibitors such as mizagliflozin [15].

## Supplementary Information

Below is the link to the electronic supplementary material.

Supplementary file1 (DOCX 5993 KB)

## Data Availability

The datasets used and/or analyzed during the current study are available from the corresponding author on reasonable request.

## Abbreviations

AGEs: Advanced glycation end-products
AMPK: AMP-activated protein kinase
EGFR: Epidermal growth factor receptor
HFD: High fat diet
LV: Left ventricle
Nox2: Nicotinamide adenine dinucleotide phosphate oxidase 2
PKC: Protein kinase C
ROS: Reactive oxygen species
SGLT1: Sodium-glucose co-transporter 1
