# Supplementary material for: Cardiac sodium-glucose co-transporter 1 (SGLT1) contributes to heart failure in a mouse model of diabetic cardiomyopathy
Source: Basic Res Cardiol. 2025 Sep 11;120(6):1193–207. doi: 10.1007/s00395-025-01136-7 (PMC12680731; doi:10.1007/s00395-025-01136-7)
Supplement: Supplementary file 1 — Supplementary file1 (DOCX 5993 KB) [file 395_2025_1136_MOESM1_ESM.docx]

**Supplementary Information (SI)**

**Cardiac Sodium-Glucose Co-Transporter 1 (SGLT1) Contributes to Heart Failure in a Mouse Model of Diabetic Cardiomyopathy**

Zhao Li, Sydney Freiberg, Meredith L. Music, Lina Gu, Sarah Nacos, Joseph P. Phillips, Adil Hassan, Kamel Shibbani, Sanah S. Munir, Vooha K. Kumar, Luke Halligan, Mia E. Michel, Benjamin F. London, Ngan Bui, Michael Cicha, Valerie Buffard, E. Dale Abel, Ferhaan Ahmad^*^

^*^ Corresponding Author:

Ferhaan Ahmad, MD, PhD, FRCPC, FACC, FAHA

Division of Cardiovascular Medicine, Department of Internal Medicine, Carver College of Medicine and Abboud Cardiovascular Research Center, University of Iowa, Iowa City, IA

E-mail: [ferhaan-ahmad@uiowa.edu](mailto:ferhaan-ahmad@uiowa.edu)

**Supplementary Figures**


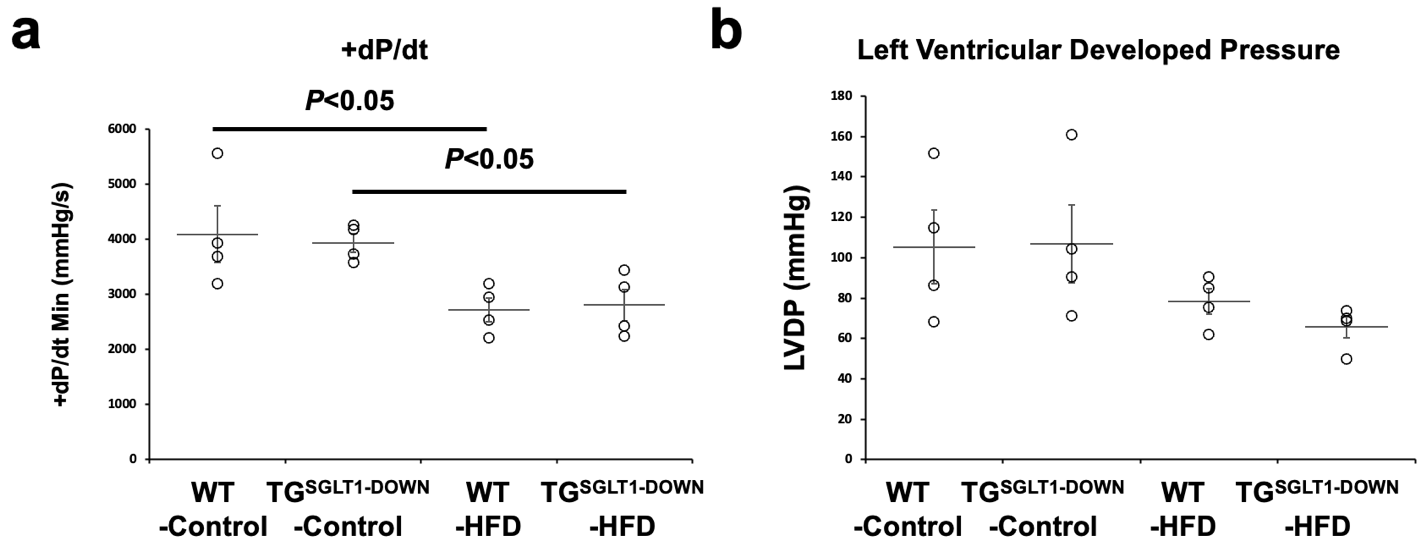


**Supplementary Fig. 1** *Ex vivo* hemodynamic studies. Eight-week-old TG^SGLT1-DOWN^ mice and WT littermates were fed a HFD or control chow for 20 weeks (*n*=4/group, 2 male and 2 female). **a** HFD caused similar decreases in +dP/dt and in WT and TG^SGLT1-DOWN^ mice. **b** Left ventricular developed pressure was not significantly different among groups. No significant sex differences were observed


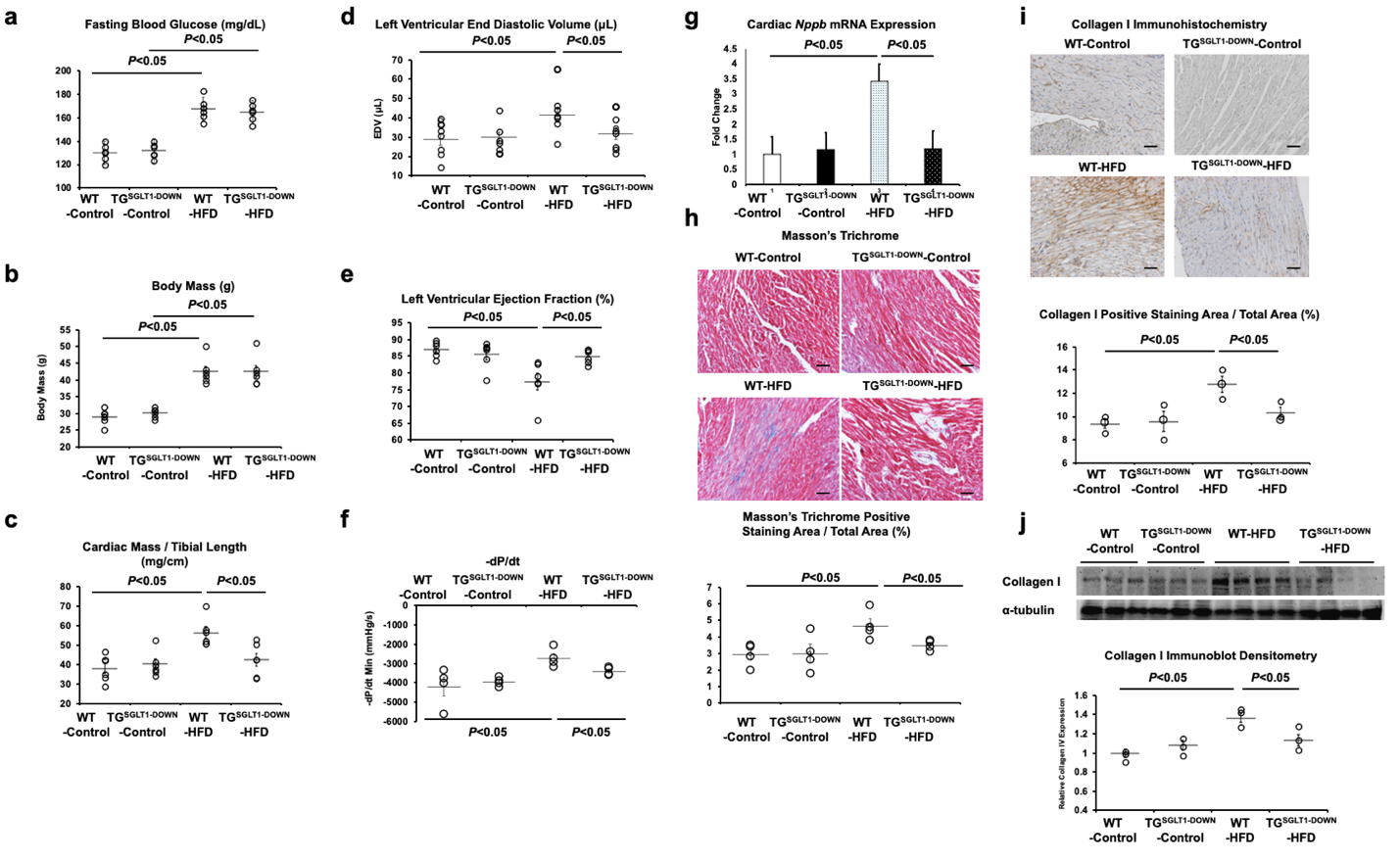


**Supplementary Fig. 2** Truncated versions of graphs from Fig. 3 for better visualization of individual data points. Transgenic knockdown of cardiomyocyte SGLT1 attenuates cardiomyopathy *in vivo*. Eight-week-old TG^SGLT1-DOWN^ mice and WT littermates were fed a HFD or control chow for 20 weeks (*n*=4-6/group, with equal numbers of males and females, as shown in the figures). HFD caused similar increases in **(a)** fasting blood glucose and **(b)** body mass in WT and TG^SGLT1-DOWN^ mice. No significant sex differences were observed. Relative to WT mice exposed to HFD, TG^SGLT1-DOWN^ mice were protected against **(c)** cardiac hypertrophy as assessed by the ratio of cardiac mass to tibial length after sacrifice, **(d)** left ventricular dilation and **(e)** deterioration in left ventricular ejection fraction (LVEF) on echocardiography before sacrifice, and **(f)** deterioration of diastolic function on *ex vivo* hemodynamic studies. TG^SGLT1-DOWN^ mice had lower cardiac expression of *Nppb* on QPCR **(g)**, less cardiac fibrosis on Masson trichrome staining **(h)**, and less cardiac expression of collagen I on immunohistochemistry **(i)** and immunoblot **(j)**. Scale bars on histology images represent 100 μm. α-tubulin was used as a loading control on the immunoblot
